# Thrombocytopenia and splenomegaly: an unusual presentation of congenital hepatic fibrosis

**DOI:** 10.1186/1750-1172-5-4

**Published:** 2010-04-12

**Authors:** Serena Botto Poala, Gianni Bisogno, Raffaella Colombatti

**Affiliations:** 1 Department of Pediatrics, University of Padova, Padova, Italy; 2 Clinic of Pediatric Hematology_Oncology, Department of Pediatrics, University of Padova, Padova, Italy

## Abstract

Congenital hepatic fibrosis (CHF) is a rare autosomal recessive disease that primarily affects the hepatobiliary and renal systems. It is characterized by hepatic fibrosis, portal hypertension, and renal cystic disease. Firm or hard hepatomegaly is present nearly in all patients, often with a prominent left lobe, and this is usually one of the presenting signs. The haematological manifestations due to hypersplenism generally arise when the other gastrointestinal manifestations are clearly developed. We describe the first case of CHF presenting in an otherwise healthy child, with thrombocytopenia and splenomegaly as the only manifestations of the disease.

## Background

Congenital hepatic fibrosis (CHF) is a rare autosomal recessive disease that primarily affects the hepatobiliary and renal systems. It is characterized by hepatic fibrosis, portal hypertension, and renal cystic disease [1-3]. CHF results from a malformation of the ductal plate (the embryological precursor of the biliary system), secondary biliary strictures, and periportal fibrosis. This subsequently results in the development of portal hypertension [4]. The exact incidence and prevalence of CHF are not known. Only a few hundred patients with CHF have been reported in the literature [1-4]. The disease appears in both sporadic and familial patterns and is associated with a wide spectrum of disorders, the most frequent of which is autosomal recessive polycystic kidney disease (ARPKD), but can also be an isolated condition [1,2].

The age at clinical onset and the spectrum of clinical manifestations is highly variable because CHF has 4 different forms: portal hypertensive (most common), cholangitic, mixed, and latent. Therefore most patients present with hepatomegaly and portal hypertension (hematemesis or melena is the presenting sign in 30 to 70% of patients) in the first decades of life and some patients present with cholangitis [1-5]. Patients with the latent form generally present at an older age or are diagnosed as an incidental finding. Firm or hard hepatomegaly is present nearly in all patients, often with a prominent left lobe, and this is usually one of the presenting signs.

The hematological manifestations due to hypersplenism generally arise when the other gastrointestinal manifestations are clearly developed [1-5].

We describe the first case of CHF presenting in an healthy child, with thrombocytopenia and splenomegaly as the only manifestations of the disease.

## Case presentation

A 10-year-old girl of Caucasian origin was admitted to our Pediatric Hematology-Oncology Outpatient Clinic for thrombocytopenia (68 × 10^3^/mm^3^) and splenomegaly. Family history was unremarkable except for the girl's mother being affected by microhematuria and asymptomatic proteinuria. The girl's past personal medical history showed asymptomatic microhematuria since age 4. Three weeks before the visit at our Clinic she had started presenting headache with an itching rubeoliform rash all over the body.

Physical examination was unremarkable except for splenomegaly (4,5 cm). The blood count showed thrombocytopenia (68 × 10^3^/mm^3^) while the remaining values were within normal range (haemoglobin 13,2 g/dL, mean corpuscular volume 75,8 fL, leukocyte 4.2 × 10^3^/mm^3^, with neutrophil 2.5 × 10^3^/mm^3^, reticolocytes 41 × 10^3^/mm^3^). Kidney and liver function test were all within normal ranges: aspartate aminotransferase 34 U/L (nv 15-40), alanine aminotransferase 24 U/L (nv 5-40), bilirubin 21,7 umol/L (nv 1,7-17), γGT 12 U/L (nv 3-22), alkaline phosphatase 177 IU/L (nv 56-247), creatinine 39 umol/L (nv 27-62).

Coagulation (PT 58%, IRN 1.42, PTT 33 sec) and haptoglobin were normal, while Coombs Test was negative.

Serological tests for hepatitis B virus, hepatitis C virus, HIV, cytomegalovirus, Epstein-Barr virus, parvovirus, Toxoplasma, Rubeovirus, Herpes Simplex virus 1-2, HHV6-7-8, Bartonella, Weil- Felix, Widal-Wright, Plasmodium Falciparum were all negative.

Peripheral blood smear and bone marrow aspirate, performed in order to exclude leukemia and metabolic diseases, were normal.

The abdominal ultrasound showed splenomegaly (17 cm) with liver and kidneys of normal size and echogenicity. A Doppler ultrasound showed a normal portal blood flow. The thorax x-Ray was normal.

Two months later, due to the persistence of the thrombocytopenia (57 × 10^3^/mm^3^), in order to exclude an autoimmune disease, we evaluated antiplatelet antibodies, Antinuclear (ANA), antiDNA, anti-extractable nuclear antigen (ENA), antimitochondrial and anti parietal cell (APCA) antibodies, anti muscle antibody which all resulted negative.

Three months after the initial evaluation, physical examination, thrombocytopenia and abdominal ultrasound remained unchanged. The child persisted in good general conditions.

Six months later, due to the persistence of both the thrombocytopenia (50 × 10^3^/mm^3^) and the splenomegaly, an abdominal CT scan was performed confirming the splenomegaly but showing also an ectasia of the portal vein, hepatomegaly -with the left liver lobe reaching the left posterior axillary region- with dilatation of the intrahepatic bile ducts, mainly in the right liver lobe.

**Figure 1:**
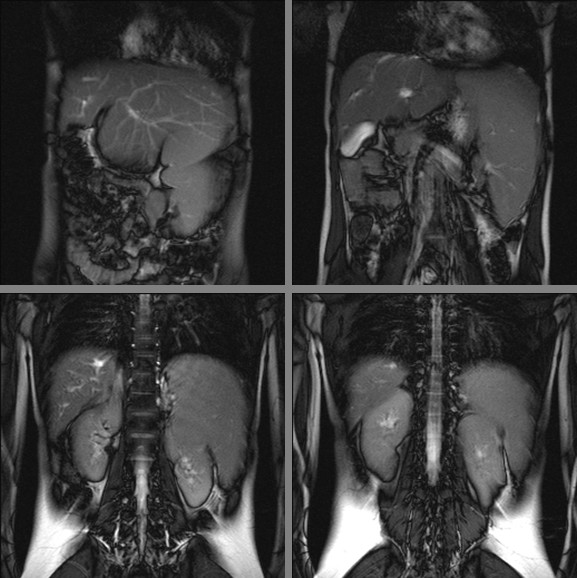
**Abdomen MRI showing reduction of the volume of the right liver lobe, hypertrophy of the lateral segment of the left liver lobe and splenomegaly**.

**Figure 2:**
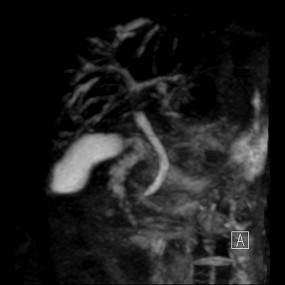
**Cholangio MRI showing slight dilatation of the biliary tract of the right lobe, normal hepatic duct and choledoch**.

Due to the results of the abdominal CT scan, the girl was admitted to the Pediatric Gastroenterology and Hepatology Unit for further investigations of a possible liver pathology. Ceruloplasmin 0,29 g/l (nv 0,22-0,6), cupremia 9,9 umol/l (nv 14-25), cupruria 0,543 umol/24 h (nv 0,047-0,55), α fetoprotein 1,3 ug/l, α1- antitrypsin 135 mg/dl were all within normal ranges. An abdomen MRI revealed reduction of the volume of the right liver lobe, hypertrophy of the lateral segment of the left liver lobe and splenomegaly (Figure 1). Cholangio-MRI revealed slight dilatation of the biliary tract of the right lobe; the hepatic duct and the choledoch were normal (Figure 2).

Hepatobiliary scintigraphy showed hepatomegaly of the left lobe, with a normal captation of the radionuclide; after the first forty-five minute there was a feeble visualization of some parts of the biliary tract and of the bowel; an elevated parenchymal activity persisted after the first hour of acquisition. After two hours from a fat meal there was an elevated bowel activity; the outflow of the left lobe was complete, while there was a persistence of the activity in the lateral portion of the right lobe. Gastroscopy revealed esophagogastric varices. The liver biopsy, performed on both the right and the left lobe, demonstrated the characteristic abnormalities of congenital hepatic fibrosis (persistence of bile ducts remnants, abnormal branching of the intrahepatic portal veins and fibrosis of the portal tracts) on both lobes; the kidney biopsy was normal.

## Discussion

CHF is a very rare disease with heterogeneous clinical manifestations [1-5].

The case that we describe is the first reported case of CHF with splenomegaly and thrombocytopenia as the only presenting signs. Hepatomegaly on physical examination has been described in all the reported cases of CHF. The absence of this sign and the normality of liver function in our patient is surprising and contributed to the delay in the diagnosis of CHF. Despite the lack of physical (hepatomegaly) and echocardiographic (increased echogenicity of the liver) signs of CHF, on MRI she displayed some of the common features of CHF[6]: abnormal large left lobe of the liver, enlarged spleen accompanied by slight dilatation of the intrahepatic bile ducts in the right lobe. Moreover, the girl showed the signs of portal hypertension, one of the prominent manifestations of CHF: splenomegaly with an ectasia of the portal vein and esophageal varices. Therefore, in our patient the suspect of primary hepatic pathology arose with the abdominal CT scan, but the final diagnosis of CHF was histopathologic, with the liver biopsy showing the typical histological abnormalities of CHF [6].

The absence of frank cystic dilatation of bile ducts on Colangio-MRI excluded Caroli's disease (a congenital disorder characterized by cystic dilatation of intrahepatic bile ducts without CHF) and Caroli's syndrome (the association of CHF and macroscopic liver cysts in continuity with the bile ducts).

The hematologic manifestations of CHF generally arise after the development of hypersplenism, secondary to portal hypertension, and include anemia, thrombocytopenia and leucopenia. To our knowledge, features of hypersplenism in children with CHF have always been associated with hepatomegaly and usually accompany other complex gastrointestinal manifestations, like bleeding from esophageal varices [7-10]. A child with mainly hematologic symptoms mimicking acute leukemia and diagnosed with CHF has been described, but he had a three-lineage cytopenia coupled with both splenomegaly and hepatomegaly [11]. Other children with pancytopenia have also been reported, but again it was associated with important hepatomegaly and alteration of hepatic function [7,10].

Our child had microhematuria since age 4 and the child's mother had microhematruria and proteinuria since 20 years of age. The child had also enlarged kidneys with normal echogenicity on abdominal ultrasound, but the kidney biopsy was normal. Isolated microhematuria and tubular defects leading to proteinuria are usually not the typical manifestations of renal involvement in CHF and other ciliopathies. In fact, CHF is an hepatic fibrocystic disease caused by defective ciliary proteins, called "ciliopathies" [12]. In ciliopathies, the liver and the kidneys are commonly affected organs, because primary cilia host critical signal transduction pathways and also sense chemical, osmotic, and mechanical stimuli such as luminal fluid flow, and regulate important cellular functions including proliferation and maintenance of planar cell polarity and mitotic spindle orientation to ensure normal epithelial function and normal diameter of tubular structures such as renal and biliary ducts. In ciliopathies, the kidneys display pathologies ranging from a urinary concentration defect in normal appearing kidneys to cystic dysplastic kidneys. Nevertheless, since an increasing number of human disorders associated with ciliary dysfunction is just being unraveled, new discrete clinical entities might be defined in the future [13]. Moreover, further follow-up of the girl and the mother might clarify the renal involvement in both of them, since renal damage can progress with aging.

Thrombocytopenia is a very common problem that pediatricians have to face in their clinical practice. It is usually an acute episode caused by increased destruction during Idiopathic Thrombocytopenic Purpura (ITP) and resolves spontaneously. When thrombocytopenia is persistent, more rare causes must be considered, whether autoimmune, metabolic or hereditary [14].

Our case demonstrates that when the most common causes of thrombocytopenia have been ruled out, other rare conditions including liver diseases must be considered. CHF must be kept in mind when splenomegaly is present even if hepatomegaly is not part of the physical examination. In our case the hypertrophy of the left hepatic lobe hepatomegaly was visible only on the CT scan, developing behind the spleen towards the left axillary region and not toward the lower abdomen. Despite the scarce symptoms and signs shown by the child, she already had esophageal varices showing a progression of the disease. Therefore, our case suggests that, in patients with persistent thrombocytopenia and splenomegaly, if autoimmune (ITP) disorders have been excluded, the abdominal CT scan might help in defining the diagnosis.

## Conclusions

In conclusion, CHF diagnosis should be considered by pediatricians in children with thrombocytopenia and splenomegaly as a rare, but still possible, cause of the signs and symptoms, even in the absence of hepatomegaly. Moreover, our case demonstrates that CHF can present in childhood with mild hematologic manifestations, without hepatomegaly on physical examination.

## Competing interests

The authors declare that they have no competing interests.

## Authors' contributions

GB clinically managed the patient, designed and interpreted the manuscript, reviewed the manuscript and gave the final approval of the version to be published. SPeLeS Project (Specializzandi in Pediatria e Letteratura Scientifica) Coordinator

SB and RC have been involved in drafting the manuscript, revising it critically for important intellectual content and approved the final version to be published

## Consent

Written informed consent was obtained from the parents of the patient for publication of this case report and any accompanying images. A copy of the written consent is available for review by the Editor-in-Chief of this journal.
